# Factors associated with breast-feeding initiation and continuation in Canadian-born and non-Canadian-born women: a multi-centre study

**DOI:** 10.1017/S1368980021004699

**Published:** 2021-12-03

**Authors:** Rishma Chooniedass, Marie Tarrant, Sarah Turner, Heidi Sze Lok Fan, Katie Del Buono, Stephanie Masina, Allan B Becker, Piushkumar Mandhane, Stuart E Turvey, Theo Moraes, Malcolm R Sears, Padmaja Subbarao, Meghan B Azad

**Affiliations:** 1 School of Nursing, Faculty of Health and Social Development, University of British Columbia, Kelowna, BC, Canada; 2 School of Nursing, Li Ka Shing Faculty of Medicine, University of Hong Kong, Hong Kong, SAR, People’s Republic of China; 3 Manitoba Interdisciplinary Lactation Centre, Winnipeg, MB, Canada; 4 Children’s Hospital Research Institute of Manitoba, Department of Pediatrics and Child Health, Max Rady College of Medicine, Rady Faculty of Health Sciences, University of Manitoba, Winnipeg, MB, Canada; 5 Department of Pediatrics, Faculty of Medicine and Dentistry, University of Alberta, Edmonton, AB, Canada; 6 Department of Paediatrics, BC Children’s Hospital Research Institute, University of British Columbia, Vancouver, BC, Canada; 7 Department of Paediatrics, University of Toronto and Hospital for Sick Children, Toronto, ON, Canada; 8 Department of Medicine, McMaster University, Hamilton, ON, Canada

**Keywords:** Breast-feeding initiation, Breast-feeding continuation, Canadian-born, Non-Canadian born, Immigrant

## Abstract

**Objective:**

To identify factors associated with breast-feeding initiation and continuation in Canadian-born and non-Canadian-born women.

**Design:**

Prospective cohort of mothers and infants born from 2008 to 2012: the Canadian Healthy Infant Longitudinal Development (CHILD) Cohort Study.

**Setting:**

General community setting in four Canadian provinces.

**Participants:**

In total, 3455 pregnant women from Vancouver, Edmonton, Winnipeg and Toronto between 2008 and 2012.

**Results:**

Of 3010 participants included in the current study, the majority were Canadian-born (75·5 %). Breast-feeding initiation rates were high in both non-Canadian-born (95·5 %) and Canadian-born participants (92·7 %). The median breast-feeding duration was 10 months in Canadian-born participants and 11 months in non-Canadian-born participants. Among Canadian-born participants, factors associated with breast-feeding initiation and continuation were older maternal age, higher maternal education, living with their partner and recruitment site. Rooming-in during the hospital stay was also associated with higher rates of breast-feeding initiation, but not continuation at 6-month postpartum. Factors associated with non-initiation of breast-feeding and cessation at 6-month postpartum were maternal smoking, living with a current smoker, caesarean birth and early-term birth. Among non-Canadian-born participants, maternal smoking during pregnancy was associated with lower odds of breast-feeding initiation and lower odds of breast-feeding continuation at 6 months, and older maternal age and recruitment site were associated with breast-feeding continuation at 6 months.

**Conclusions:**

Although Canadian-born and non-Canadian-born women in the CHILD cohort have similar breast-feeding initiation rates, breast-feeding initiation and continuation are more strongly associated with socio-demographic characteristics in Canadian-born participants. Recruitment site was strongly associated with breast-feeding continuation in both groups and may indicate geographic disparities in breast-feeding rates nationally.

Breast-feeding has multiple health benefits for both mothers and children, including maternal protection against breast and ovarian cancer, as well as Type 2 diabetes^(1)^. Children who are breastfed have increased intelligence, protection against infections and reductions in the prevalence of asthma, allergies, obesity and diabetes^(2–4)^. In addition to the multiple physical health benefits, maternal–infant bonding during breast-feeding promotes security and emotional stability for the child^(5)^.

In Canada, 89 % of women initiate breast-feeding, but only 26 % exclusively breastfeed to 6 months, despite the WHO recommendations for a minimum duration of 6 months^(6)^. Early breast-feeding cessation is related to many different factors, including socio-demographic characteristics such as younger maternal age, lower education and family income, returning to work and maternal smoking^(7–9)^; hospital practices such as delayed initiation of breast-feeding and separation of the mother and infant and intrapartum and birth factors such as caesarean section and gestational age^(10–13)^. Because of cultural practices, the country of birth can also play a critical role in breast-feeding. Women who live in low- and middle-income countries^(14)^ generally breastfeed their children for longer when compared with women in high-income countries^(2)^. Among immigrant women in high-income countries, maternal ethnicity and birth country have been shown to influence breast-feeding practices^(15)^. Within countries, race and ethnicity are associated with the rate of breast-feeding, with Caucasian Americans usually having a higher rate of breast-feeding when compared with African Americans^(16)^.

A recent review of breast-feeding practices among immigrant and non-immigrant women by Dennis *et al.*^(17)^ reported that immigrant women were twice as likely to breastfed for 3–6 months and were 33 % more likely to breastfeed for longer than 6-month postpartum. Studies also show that although immigrant women have higher breast-feeding rates when compared with native-born women, these rates decrease with the length of time that the immigrant lives in the country^(18)^. However, one study showed that in Spain, native-born women were more likely to exclusively breastfeed at the time of postpartum hospital discharge when compared with Chinese immigrant women^(19)^. Although the USA and Canada are culturally diverse, immigrants in both countries tend to assimilate to a new culture for social acceptance. Immigrant women often feel conflicted between family and cultural practices and the accepted practices in a new country^(20)^. Additionally, age at the time of migration and the country where women receive their education can influence the way they adapt and can be a strong predictor of breast-feeding initiation and duration^(21)^.

Over the last several decades, there have been increased efforts to promote and encourage breast-feeding in Canada. Immigration is also on the rise, especially for women of childbearing age^(22)^. Non-Canadian-born residents now account for 20 % of the Canadian population^(23)^. Few studies have assessed how patterns of immigration and the length of time spent in Canada affect immigrant women’s breast-feeding behaviours and how this compares with Canadian-born women^(24)^. Therefore, the current study aimed to compare breast-feeding initiation and continuation rates among Canadian-born and non-Canadian-born women and to examine factors associated with breast-feeding initiation and continuation among both groups.

## Methods

### Design, setting and participants

**Fig. 1:**
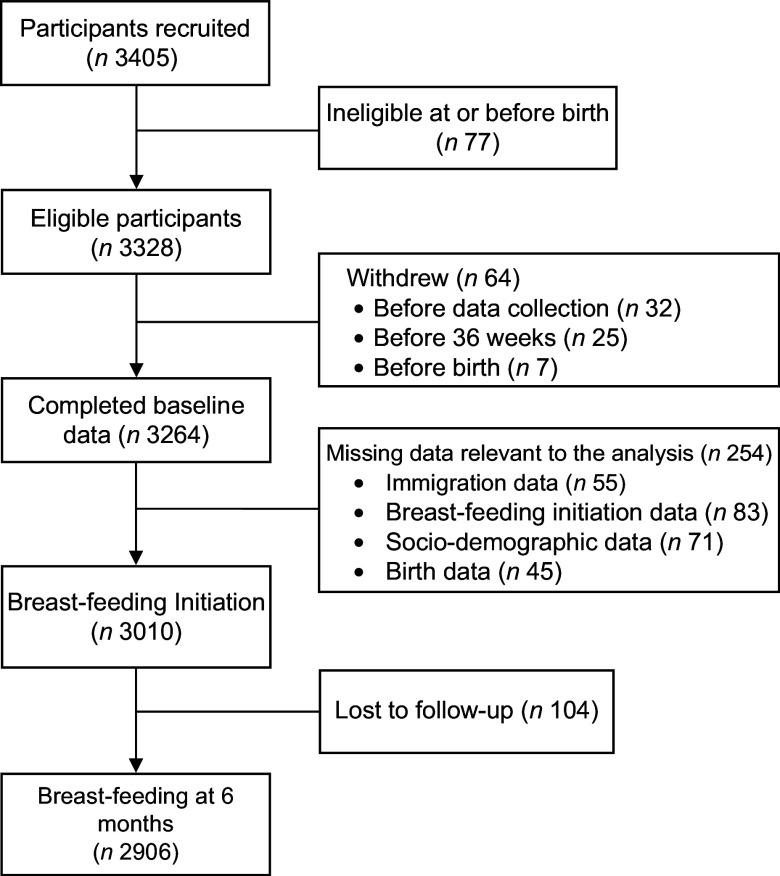
Participant flow diagram

We accessed data from the CHILD Cohort Study, a multi-center national birth cohort study that recruited pregnant women from four Canadian provinces. From 2008 to 2012, the study recruited 3621 participants in their second and third trimester, and prospective follow-up is still ongoing^(25)^. At the time of the study, only one recruitment site was designated as a Baby-Friendly Hospital^(26)^. The present analysis excluded participants from the Vanguard cohort and only focused on the general cohort of 3405 participants (see Fig. 1). Among these, seventy-seven infants were ineligible at or before birth, 64 mothers subsequently withdrew and 254 were missing relevant breast-feeding data. Complete baseline and breast-feeding initiation data were available for 3010 participants. There was a small loss to follow-up (*n* 104) with 6-month breast-feeding status was available for 2906 of these participants. A comparison of the demographic characteristics of those with full follow-up and those with missing data or who were lost to follow-up at 6 months shows that participants with missing date were more likely to be younger, have lower education and poorer socio-economic status (see online supplementary material, Supplemental Table S1). There were fewer observed differences between participants with full follow-up and those missing only the 6-month follow-up data. Eligibility criteria for the CHILD study included pregnant women aged 18 and older (19 in Vancouver) with a singleton pregnancy. Women were excluded if they used *in vitro* fertilization to conceive, gave birth before 35 weeks’ gestation or were unable to speak and read English. Recruitment methods included referrals from physicians and midwives, community and social media advertising and word of mouth.

### Data collection procedures

All participants signed written consent before participation in the study. Participants completed baseline socio-demographic questionnaires that recorded ethnicity, country of birth, years lived in Canada, education and socio-economic status. Detailed questionnaires were completed prenatally and at 3, 6, 12, 18 and 24 months postpartum. Participants were asked about breast-feeding initiation and duration. Hospital information (gestational age, birth mode, feeding and birth weight) was collected by hospital labour and delivery nurses and research staff.

### Study variables

Breast-feeding initiation was recorded in the postpartum period on the hospital records and was extracted by the study research staff. Continuing breast-feeding status was self-reported on the follow-up questionnaires at multiple intervals after delivery (3, 6, 9, 12, 18 and 24 months). In these questionnaires, we assessed breast-feeding initiation, breast-feeding status at 6-month postpartum and total duration of any breast-feeding. Any breast-feeding was defined as the receipt of any breast milk, either at the breast or expressed, irrespective of the concurrent receipt of infant formula or other breast milk substitutes^(27)^.

Immigration status variables were extracted from the demographic questions on country of birth, age at the time of migration and length of time lived in Canada. The country of birth was used to determine the geographic region, country income level and gender-related development index. The geographic region of birth and country income level were categorised according to data from the World Bank^(28)^. Gender Development Index is a measurement created by the UN to measure gender inequalities in three dimensions of human development for females as compared with males: life expectancy at birth, years of education and estimated earned income^(29)^.

We also included socio-demographic and maternal and birth characteristics that have been shown to affect breast-feeding in most populations. Socio-demographic characteristics that were included in the analysis were maternal age, maternal education, household income, home ownership, marital status, recruitment site and maternal and household smoking, all self-reported at enrolment. Maternal and birth characteristics included parity, mode of birth, gestational age, birth weight and rooming-in, all extracted from the postpartum hospital record.

### Statistical analysis

**Fig. 2:**
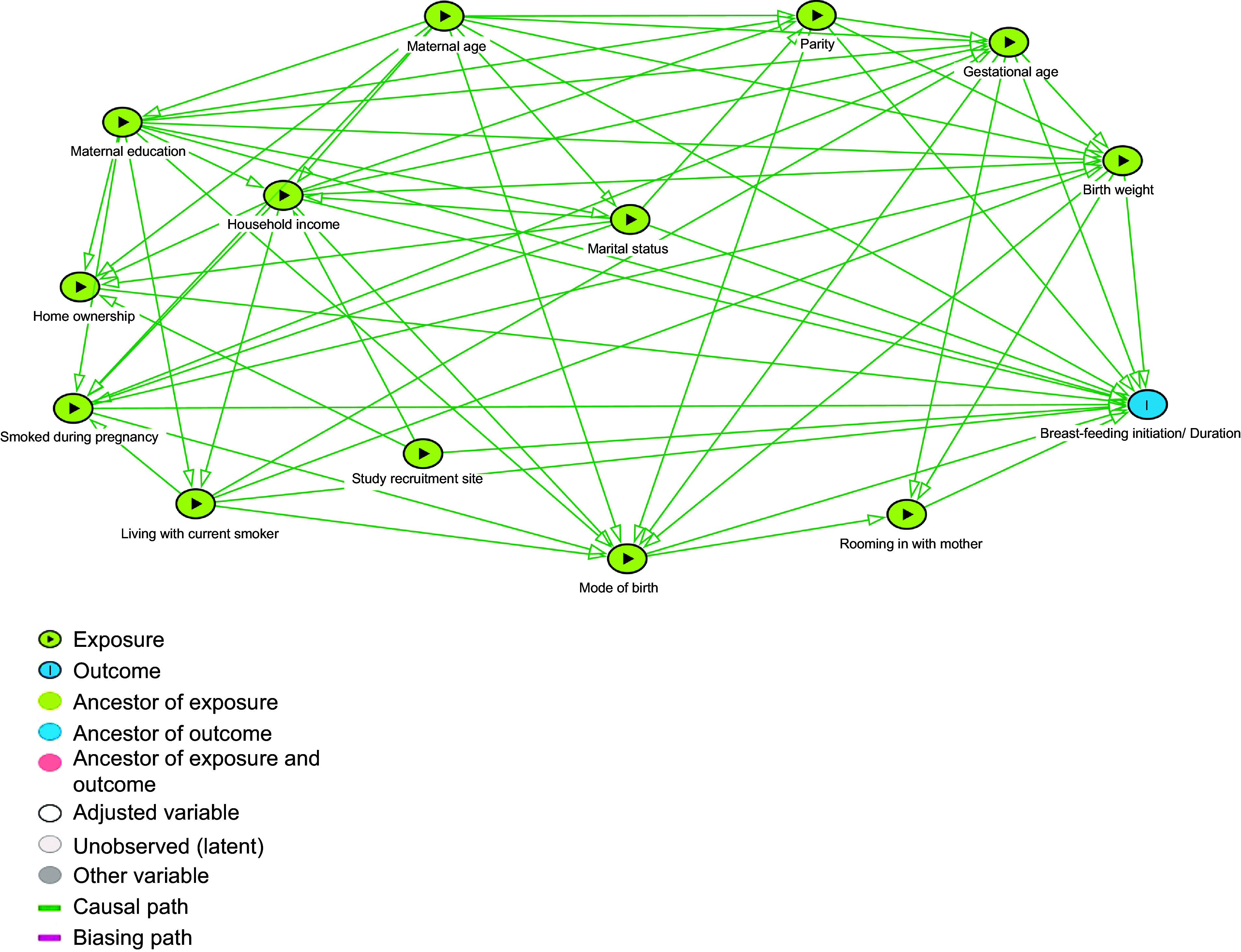
Directed acyclic graph of the explanatory model of the factors related to breast-feeding outcomes

We used descriptive statistics to summarise the socio-demographic characteristics and birth-related factors of Canadian-born and non-Canadian-born participants. Bivariable and multivariable logistic regression analysis was used to assess the association between place of birth and breast-feeding initiation and continuation at 6 months as well as factors associated with breast-feeding in Canadian-born participants and non-Canadian-born participants. Figure 2 presents the directed acyclic graph of the explanatory model^(30)^. The directed acyclic graph was used to determine the minimum set of covariates required for adjustment when each variable was set as the exposure. Hosmer–Lemeshow tests were used to test the goodness of fit. Data were analysed using Stata version 15.1^(31)^. A 95 % CI and 0·05 level of significance were used for analysis.

## Results

Table 1 shows the characteristics of Canadian-born and non-Canadian-born participants. Of 3010 participants included in the current study, the majority were Canadian-born (75·4 %) with a university degree or higher (62·9 %). Although a higher proportion of non-Canadian-born participants (69·2 %) had a university degree when compared with Canadian-born participants (60·9 %), household income was lower in non-Canadian-born participants. Among non-Canadian-born participants, approximately one-half lived in Canada for < 10 years, 63·1 % migrated when they were 18 years of age or older and 63·3 % lived in Vancouver or Toronto. Around 40 % of non-Canadian-born participants were born in South & East Asia & Pacific Region and almost one-half were from a country with a high Gender Development Index.

**Table 1:** A comparison of the characteristics of immigrant and Canadian-born study participants (*n* 3010)

Characteristic	Canadian-born *n* (%) 2271 (75·4)		Non-Canadian born *n* (%) 739 (24·6)		*P*-value
	*n*	%	*n*	%	
Socio-demographic characteristics					
Maternal age (years)					0·002
18–24	166	7·3	33	4·5	
25–29	513	22·6	158	21·4	
30–34	981	43·2	306	41·4	
≥ 35	611	26·9	242	32·8	
Maternal education					< 0·001
High-school or below	206	9·1	48	6·5	
Some postsecondary	683	30·1	180	24·4	
University degree or above	1382	60·9	511	69·2	
Annual household income (CAD)					< 0·001
< $50 000	234	10·3	140	18·9	
$50 000–$99 999	671	29·6	239	32·3	
$100 000–$149 999	628	27·7	141	19·1	
≥ $150 000	532	23·4	141	19·1	
Not specified	206	9·1	78	10·6	
Home ownership					< 0·001
No	503	22·2	280	37·9	
Yes	1768	77·9	459	62·1	
Married or living with partner					.16
No	146	6·4	37	5·0	
Yes	2125	93·6	702	95·0	
Study recruitment site					< 0·001
Edmonton	559	24·61	124	16·8	
Toronto	508	22·37	215	29·1	
Vancouver	430	18·93	253	34·2	
Winnipeg	774	34·08	147	19·9	
Smoked during pregnancy					< 0·001
No	2169	95·5	728	98·5	
Yes	102	4·5	11	1·5	
Living with current smoker					0·03
No	1966	86·6	663	89·7	
Yes	305	13·4	76	10·3	
Maternal and birth characteristics					
Parity					0·36
Primiparous	1167	51·4	394	53·3	
Multiparous	1104	48·6	345	46·7	
Mode of birth					0·02
Vaginal	1718	75·7	528	71·5	
Caesarean section	553	24·4	211	28·6	
Gestational age					.19
Preterm (35–< 37 weeks)	24	3·3	98	4·3	
Early-term (37–< 39 weeks)	186	25·2	513	22·6	
Full-term (≥ 39 weeks)	529	71·6	1660	73·1	
Birth weight (g)					< 0·001
< 2500	46	2·0	25	3·4	
2500–< 3250	652	28·7	260	35·2	
3250–< 4000	1243	54·7	375	50·7	
≥ 4000	330	14·5	79	10·7	
Baby rooming in with mother					0·05
No	372	16·4	99	13·4	
Yes	1899	83·6	640	86·6	
Initiated breastfeeding					0·007
No	166	7·3	33	4·5	
Yes	2105	92·7	706	95·5	
Breast-feeding at 6 months					0·002
No	549	24·8	132	19·1	
Yes	1665	75·2	560	80·9	
Immigration characteristics					
Years lived in Canada					
< 5			213	28·8	
5–< 10			158	21·4	
10–< 20			154	20·8	
≥ 20			214	29·0	
Mother’s age at migration					
< 18	–		237	36·9	
≥ 18	–		466	63·1	
Economic region					
North America	–		71	9·6	
Latin America & Caribbean	–		105	14·2	
Europe & Central Asia	–		191	25·9	
Middle East & Africa	–		74	10·0	
Asia & Pacific	–		298	40·3	
Country income level					
High	–		325	44·0	
Upper middle	–		198	26·8	
Lower middle – low	–		216	29·2	
GDI					
High	–		354	47·9	
Moderate	–		279	37·8	
Low	–		106	14·3	

GDI: Gender Development Index.

The median breast-feeding duration was 10 months in Canadian-born participants and 11 months in non-Canadian-born participants (*P* < 0·001). Participants who migrated before 18 years of age had the same median duration of breast-feeding as Canadian-born participants (10 months), while participants who migrated after 18 years of age had a median duration of breast-feeding of 11 months. When compared with Canadian-born participants, non-Canadian-born participants were more likely to initiate breast-feeding (95·5 % *v*. 92·7 %; *P* = 0·007) and to continue breast-feeding at 6 months (80·9 % *v*. 75·2 %; *P* = 0·002) in unadjusted analyses (Table 1). After adjusting for all socio-demographic and labour and birth factors, however, these differences were no longer significant for breast-feeding initiation (OR = 0·76; 95 % CI: 0·50, 1·14) or for breast-feeding continuation at 6 months (OR = 0·92; 95 % CI: 0·72, 1·16) (data not shown).

Table 2 shows the unadjusted and adjusted OR for associations between the socio-demographic, labour and birth factors and breast-feeding initiation among Canadian-born participants and non-Canadian-born participants. Canadian-born participants were more likely to initiate breast-feeding if they were older, had some post-secondary education, were married or living with their partner and were rooming-in during the hospital stay (all *P* < 0·05). In non-Canadian-born participants, older maternal age and higher education were also associated with breast-feeding initiation, but the association was only significant in participants 30–34 years of age and those with a university degree or above. When compared with participants from other centres, Canadian-born participants recruited in Vancouver were more likely to initiate breast-feeding, but there were no significant differences in breast-feeding initiation by study site among non-Canadian-born participants. Maternal smoking was associated with lower odds of breast-feeding initiation in non-Canadian-born participants, while living with a current smoker, caesarean birth and early-term birth (37–< 39 weeks) were associated with lower odds of breast-feeding initiation in Canadian-born participants.

**Table 2:** Unadjusted and adjusted odds ratios for breast-feeding initiation by birthplace (*n* 3010)

	Canadian-born (*n* 2271)				Non-Canadian born (*n* 739)			
	Unadjusted		Adjusted*		Unadjusted		Adjusted*	
Characteristic	OR	95 % CI	OR	95 % CI	OR	95 % CI	OR	95 % CI
Socio-demographic characteristics								
Maternal age (years)								
18–24	1	–	–	–	1	–	1	–
25–29	2·89	1·77, 4·72	–	–	1·48	0·38, 5·70	–	–
30–34	3·81	2·42, 6·03	–	–	4·27	1·05, 17·4	–	–
≥ 35	5·36	3·14, 9·15	–	–	1·76	0·47, 6·54	–	–
Maternal education								
High-school or below	1	–	1	–	1	–	1	–
Some post-secondary	2·64	1·73, 4·04	2·20	1·41, 3·44	1·69	0·61, 4·67	1·67	0·54, 5·13
University degree or above	5·71	3·74, 8·72	4·15	2·56, 6·73	5·47	1·98, 15·1	5·27	1·66, 16·8
Annual household income (CAD)								
< $50 000	1	–	1	–	1	–	1	–
$50 000-$99 999	2·06	1·32, 3·22	1·01	0·61, 1·68	1·55	0·58, 4·11	1·31	0·47, 3·66
$100 000-$149 999	4·18	2·48, 7·04	1·59	0·86, 2·95	2·08	0·61, 7·06	1·38	0·37, 5·18
≥ $150 000	3·38	2·01, 5·67	1·11	0·57, 2·16	2·08	0·61, 7·06	1·38	0·34, 5·59
Not specified	1·96	1·08, 3·57	1·37	0·73, 2·57	0·53	0·19, 1·47	0·47	0·16, 1·42
Home ownership								
No	1	–	1	–	1	–	1	–
Yes	1·57	1·11, 2·23	1·05	0·68, 1·61	1·39	0·69, 2·80	1·37	0·61, 3·07
Married or living with partner								
No	1	–	1	–	1	–	1	–
Yes	3·78	2·44, 5·86	2·02	1·24, 3·29	1·98	0·57, 6·80	1·26	0·34, 4·61
Smoked during pregnancy								
No	1	–	1	–	1	–	1	–
Yes	0·23	0·14, 0·37	0·72	0·40, 1·29	0·11	0·03, 0·45	0·17	0·04, 0·80
Living with current smoker								
No	1	–	1	–	1	–	1	–
Yes	0·29	0·20, 0·41	0·45	0·31, 0·66	0·33	0·14, 0·77	0·48	0·20, 1·17
Study recruitment site								
Edmonton	1	–	–	–	1	–	1	–
Toronto	1·22	0·77, 1·94	–	–	0·79	0·29, 2·13	–	–
Vancouver	7·44	2·93, 18·9	–	–	2·09	0·66, 6·63	–	–
Winnipeg	0·74	0·50, 1·08	–	–	0·88	0·30, 2·62	–	–
Maternal and birth characteristics								
Parity								
Primiparous	1	–	1	–	1	–	1	–
Multiparous	0·85	0·62, 1·16	0·80	0·57, 1·13	0·93	0·46, 1·86	1·28	0·59, 2·79
Mode of birth								
Vaginal	1	–	1	–	1	–	1	–
Caesarean	0·78	0·55, 1·10	0·68	0·47, 0·99	0·79	0·38, 1·66	1·03	0·45, 2·39
Gestational age								
Full-term (≥39 weeks)	1	–	1	–	1	–	1	–
Early-term (37–< 39 weeks)	0·69	0·49, 0·98	0·67	0·47, 0·97	0·50	0·24, 1·02	0·49	0·23, 1·07
Preterm (35–< 37 weeks)	1·71	0·62, 4·75	1·70	0·60, 4·77	0·86	0·11, 6·68	0·89	0·10, 7·69
Birth weight (grams)								
< 2500	1	–	1	–	1	–	1	–
2500–< 3250	0·29	0·04, 2·17	0·40	0·05, 3·08	3·14	0·62, 16·0	4·35	0·74, 25·5
3250–< 4000	0·26	0·04, 1·90	0·34	0·04, 2·64	1·72	0·38, 7·89	1·98	0·36, 11·0
> 4000 g	0·33	0·04, 2·49	0·40	0·05, 3·27	1·06	0·20, 5·61	1·12	0·16, 7·72
Baby rooming in with mother								
No	1	–	1	–	1	–	1	–
Yes	1·70	1·17, 2·47	1·84	1·26, 2·69	0·41	0·10, 1·72	0·37	0·08, 1·60

* Covariates for each model are as follows:

Maternal age: no adjustment needed.

Maternal education: adjusted for maternal age.

Household income: adjusted for maternal age, education, marital status and study site.

Home ownership: adjusted for maternal age, education, marital status, household income, study site.

Marital status: adjusted for maternal age and education.

Smoked during pregnancy: adjusted for maternal age, education, marital status, household income and live with current smoker.

Live with current smoker: adjusted for household income and maternal education.

Study recruitment site: no adjustment needed.

Parity: adjusted for maternal age, education, marital status and household income.

Mode of birth: adjusted for maternal age, education, marital status, household income, smoked during pregnancy, live with a current smoker, parity, gestational age and birth weight.

Gestational age: adjusted for maternal age, education, household income, smoked during pregnancy, live with current smoker and parity.

Birth weight: adjusted for maternal age, education, household income, smoked during pregnancy, live with current smoker, parity and gestational age.

Baby rooming in with mother: adjusted for mode of birth, gestational age and birth weight.

The median breast-feeding duration was 12 months in Vancouver participants, 10 months in Winnipeg participants and 9 months in both Edmonton and Toronto participants (*P* < 0·001). The unadjusted and adjusted associations between the predictors of breast-feeding continuation at 6-month postpartum in Canadian-born participants and non-Canadian-born participants are shown in Table 3. In Canadian-born participants, older maternal age, post-secondary education and married or living with a partner were significantly associated with breast-feeding continuation at 6-month postpartum in the adjusted analyses, whereas in non-Canadian-born participants, only maternal age was associated with breast-feeding continuation. In both groups, participants recruited in Vancouver had significantly higher odds of breast-feeding continuation at 6-month postpartum, when compared with participants recruited in Edmonton. Maternal smoking was significantly associated with lower odds of breast-feeding continuation at 6-month postpartum in both Canadian-born (OR = 0·47; 95 % CI 0·29, 0·76) and non-Canadian-born participants (OR = 0·08; 95 % CI 0·02, 0·42). Living with a current smoker (OR = 0·49; 95 % CI 0·37, 0·64), having a caesarean section (OR = 0·62; 95 % CI 0·49, 0·79) and early-term birth (OR = 0·65; 95 % CI 0·51, 0·82) were associated with lower odds of breast-feeding continuation at 6 months in Canadian-born participants only. The results of the Hosmer–Lemeshow goodness-of-fit tests for the logistic regression models were all > 0·05, indicating that the models fit the data.

**Table 3:** Unadjusted and adjusted odds ratios for any breast-feeding at 6-month postpartum by birthplace (*n* 2906)

	Canadian Born (*n* 2214)				Non-Canadian Born (*n* 692)			
	Unadjusted		Adjusted*		Unadjusted		Adjusted*	
Characteristic	OR	95 % CI	OR	95 % CI	OR	95 % CI	OR	95 % CI
Socio-demographic characteristics								
Maternal age (years)								
18–24	1	–	–	–	1	–	–	–
25–29	4·01	2·77, 5·80	–	–	3·31	1·45, 7·57	–	–
30–34	4·97	3·51, 7·03	–	–	3·28	1·52, 7·08	–	–
≥ 35	7·36	5·03, 10·8	–	–	2·81	1·29, 6·12	–	–
Maternal education								
High-school or below	1	–	1	–	1	–	1	–
Some post-secondary	2·20	1·60, 3·02	1·73	1·23, 2·42	1·16	0·57, 2·38	0·90	0·41, 2·01
University degree or above	5·30	3·88, 7·22	3·52	2·49, 4·97	2·76	1·39, 5·47	2·11	0·95, 4·66
Annual household income (CAD)								
< $50 000	1	–	1	–	1	–	1	–
$50 000-$99 999	2·37	1·72, 3·25	1·16	0·81, 1·67	1·28	0·75, 2·20	1·12	0·63, 2·01
$100 000-$149 999	2·83	2·04, 3·92	1·05	0·70, 1·55	1·12	0·61, 2·06	0·73	0·37, 1·45
≥ $150 000	2·99	2·13, 4·19	0·96	0·62, 1·47	1·31	0·70, 2·43	0·88	0·43, 1·82
Not specified	2·00	1·33, 2·99	1·34	0·86, 2·09	1·03	0·50, 2·10	1·15	0·53, 2·47
Home ownership								
No	1	–	1	–	1	–	1	–
Yes	1·44	1·15, 1·80	1·00	0·76, 1·32	1·43	0·98, 2·11	1·55	0·996, 2·40
Married or living with partner								
No	1	–	1	–	1	–	1	–
Yes	3·77	2·68, 5·32	2·01	1·37, 2·95	2·45	1·15, 5·26	1·78	0·80, 3·97
Smoked during pregnancy								
No	1	–	1	–	1	–	1	–
Yes	0·18	0·12, 0·27	0·47	0·29, 0·76	0·06	0·01, 0·26	0·08	0·02, 0·42
Living with current smoker								
No	1	–	1	–	1	–	1	–
Yes	0·33	0·25, 0·42	0·49	0·37, 0·64	0·49	0·29, 0·84	0·61	0·35, 1·08
Study recruitment site								
Edmonton	1	–			1	–	–	–
Toronto	1·23	0·93, 1·63			1·43	0·84, 2·43	–	–
Vancouver	3·27	2·29, 4·68			2·38	1·37, 4·13	–	–
Winnipeg	0·93	0·73, 1·18			1·46	0·82, 2·60	–	–
Maternal and birth characteristics								
Parity								
Primiparous	1	–	1	–	1	–	1	–
Multiparous	1·10	0·90, 1·33	1·03	0·84, 1·28	0·87	0·59, 1·27	0·99	0·65, 1·50
Mode of birth								
Vaginal	1	–	1	–	1	–	1	–
Caesarean	0·69	0·56, 0·86	0·62	0·49, 0·79	0·81	0·54, 1·23	0·84	0·54, 1·32
Gestational age								
Full-term (≥ 39 weeks)	1	–	1	–	1	–	1	–
Early-term (37–< 39 weeks)	0·68	0·55, 0·85	0·65	0·51, 0·82	0·71	0·47, 1·08	0·76	0·49, 1·18
Preterm (35–< 37 weeks)	1·01	0·62, 1·65	1·03	0·61, 1·74	1·07	0·36, 3·22	1·33	0·40, 4·44
Birth Weight (g)								
< 2500	1		1	–	1	–	1	–
2500–< 3250	0·99	0·49, 2·01	1·08	0·50, 2·32	0·79	0·22, 2·78	0·89	0·24, 3·35
3250–< 4000	0·98	0·49, 1·96	0·96	0·44, 2·08	0·50	0·15, 1·72	0·52	0·14, 1·96
> 4000	0·96	0·47, 1·99	0·87	0·38, 1·96	0·60	0·16, 2·31	0·60	0·14, 2·56
Baby rooming in with mother								
No	1	–	1	–	1	–	1	–
Yes	1·14	0·89, 1·47	1·17	0·90, 1·52	0·82	0·46, 1·45	0·83	0·47, 1·49

* Covariates for each model are as follows:

Maternal age: no adjustment needed.

Maternal education: adjusted for maternal age.

Household income: adjusted for maternal age, education, marital status and study site.

Home ownership: adjusted for maternal age, education, marital status, household income and study site.

Marital status: adjusted for maternal age and education.

Smoked during pregnancy: adjusted for maternal age, education, marital status, household income and live with current smoker.

Live with current smoker: adjusted for household income and maternal education.

Study recruitment site: no adjustment needed.

Parity: adjusted for maternal age, education, marital status and household income.

Mode of birth: adjusted for maternal age, education, household income, smoked during pregnancy, live with a current smoker, parity, gestational age and birth weight.

Gestational age: adjusted for maternal age, education, household income, smoked during pregnancy, live with current smoker and parity.

Birth weight: adjusted for maternal age, education, household income, smoked during pregnancy, live with current smoker, parity and gestational age.

Baby rooming in with mother: adjusted for mode of birth, gestational age and birth weight.

We found no association between any of the assessed migration characteristics and breast-feeding initiation or breast-feeding continuation at 6-month postpartum in non-Canadian-born participants (see online supplementary material, Supplemental Table S2), although power was limited for the current analysis. As a sensitivity analysis, we also repeated our analysis on the predictors of breast-feeding at 6 months on a restricted subset of participants, including only participants who had initiated breast-feeding (*n* 2707). Findings from the restricted subset showed similar associations between participant characteristics and breast-feeding outcomes and the models showed similar point estimates (see online supplementary material, Supplemental Table S3).

## Discussion

This large prospective study identified factors associated with breast-feeding initiation and continuation in Canadian-born and non-Canadian-born women. Approximately one-quarter of the sample were non-Canadian-born participants, but contrary to other studies,^(17,18)^ we found only minimal differences in the breast-feeding duration of Canadian-born and non-Canadian-born women. We also did not find that migration characteristics affected either breast-feeding initiation or breast-feeding continuation in non-Canadian-born participants, although study power may have been limited for the current analysis. Multivariable analyses indicate that different factors affect breast-feeding initiation and continuation in the two groups of participants. In Canadian-born participants, socio-demographic characteristics, such as older maternal age, higher education, and living with a partner, were positively associated with breast-feeding initiation and continuation, while living with a smoker was negatively associated with both breast-feeding outcomes. These associations were observed less consistently in non-Canadian-born participants. Maternal smoking decreased the odds of breast-feeding initiation in Canadian-born participants and decreased the odds of breast-feeding continuation in all participants. In Canadian-born participants, those recruited in Vancouver had breast-feeding initiation rates that were higher than participants recruited at the other study sites. Among all participants, those recruited in Vancouver had approximately two to three times higher odds of continuing breast-feeding at 6-month postpartum. Early-term and caesarean birth were associated with lower rates of breast-feeding initiation and continuation in Canadian-born participants only.

The association between socio-demographic characteristics (e.g. maternal age, education and marital status) and breast-feeding initiation and duration has been a common finding in other studies^(33,34)^. However, in our study, the associations between breast-feeding and maternal age and education more consistent among Canadian-born women, which is similar to previous Canadian data showing that women who do not breastfeed are more likely to be younger and have less education than those who do breastfeed^(6)^. Non-Canadian-born women in our sample were more likely than Canadian-born women to have a university degree or above (69·2 % *v*. 60·9 %) and to initiate breast-feeding (95·5 % *v*. 92·7 %). Therefore, it is possible that among non-Canadian-born participants, cultural norms are more influential than socio-demographic characteristics. Studies in other countries have shown that breast-feeding practices are less associated with socio-demographic characteristics than in Western countries, and breast-feeding is sometimes more prevalent in women with lower education levels^(32,35–37)^.

Our findings on the differences in breast-feeding rates between study sites are consistent with national data reporting that British Columbia has the highest breast-feeding rates across Canada^(6)^. Breast-feeding rates in Canada vary substantially across the country, with an east-to-west gradient of lower to higher initiation rates ranging from 57 % in Newfoundland and Labrador to 96 % in British Columbia^(38)^. Furthermore, participants recruited at the Vancouver study-site were all recruited from a large tertiary hospital that was a designated Baby-Friendly Hospital Initiative (BFHI) facility, the only study site to have received that designation at the time of recruitment. Studies show that exposure to BFHI practices improves breast-feeding initiation and duration^(39,40)^, but further research is needed to explain why these disparities in breast-feeding practices continue within Canada.

Negative associations between maternal and household smoke exposure and breast-feeding duration have been consistently reported in previous studies^(7,41)^. Smoking during breast-feeding lowers prolactin levels, reduces the volume of breast milk and alters the composition and protective properties of breast milk^(42,43)^. Women who smoke or who live in a smoking household are also less likely to intend to and initiate breast-feeding^(44)^, indicating that psychosocial factors are also associated with the lower rates of breast-feeding among smoking mothers^(45)^. Also, research has found that when compared with non-smoking partners, fathers who smoke are more likely to prefer infant formula feeding over breast-feeding^(7)^, and a father’s infant feeding preferences are strongly associated with breast-feeding initiation and duration^(46,47)^.

In the literature, the associations between breast-feeding and maternal and birth factors, such as early-term birth, caesarean section and rooming-in, are inconsistent^(39,48,49)^. Participants in our study with early-term birth were 30–50 % less likely to initiate breast-feeding, and Canadian-born participants with early-term birth were 35 % less likely to continue breast-feeding at 6 months. Women giving birth early-term are more likely to have caesarean section^(50)^, and their infants are more likely to be admitted to the neonatal intensive care unit^(51)^ and to have a prolonged hospitalisation^(52)^, all of which can interfere with breast-feeding initiation and duration. A review of 53 studies concluded that caesarean section reduced the odds of breast-feeding initiation by over 40 % but had no effect on breast-feeding duration in women who initiated breast-feeding^(48)^. However, our analysis showed that caesarean birth remained significantly associated with lower odds of breast-feeding continuation at 6 months in Canadian-born participants, even among the subset of participants who initiated breast-feeding (see online supplementary material, Supplemental Table S3).

Studies in other countries have shown that immigrant women tend to breastfeed longer than native-born women^(18,53)^ and that breast-feeding duration decreases with longer time since migration^(18,54,55)^. A recent meta-analysis of twenty-nine studies found that while immigrant women were marginally more likely to initiate breast-feeding than non-immigrant women, they were twice as likely to continue breast-feeding for 12 to 24 weeks^(17)^. However, more than one-half of the included studies (16/29) did not adjust for key socio-demographic variables, such as maternal age, education and family income. In our study, we found no significant association between migration status and migration characteristics and breast-feeding initiation or continuation and other studies in Canada have also shown conflicting findings. In one study of Canadian-born and immigrant women, Dennis *et al.*^(56)^ found that migrant women were less likely to be breast-feeding exclusively at 16 weeks compared with Canadian-born women. As previously discussed, Canadian-born and non-Canadian-born participants in our study had high maternal education levels, possibly contributing to the similarity of breast-feeding rates. Furthermore, one-third of our non-Canadian-born participants immigrated to Canada before 18 years of age, possibly making them more acculturated to the Canadian lifestyle.

The current study used a large prospective national cohort with detailed data collection at numerous time points throughout the first several years of life. The dropout rate was low, and breast-feeding follow-up data were collected on 88·2 % of the sample. However, study participants had higher maternal education levels and socio-economic status when compared with the general Canadian population^(57)^, which may account for the high breast-feeding initiation rates and fewer differences between Canadian-born and non-Canadian-born participants than shown in other studies. Therefore, these findings may not be generalisable to the larger population of breast-feeding women. High breast-feeding initiation rates may have affected our ability to determine factors affecting initiation. Another limitation was the exclusion of participants who did not speak and read English as this could have excluded newly arrived immigrants who may be less acculturated to Canadian society and have higher breast-feeding rates. In addition, participants without follow-up breast-feeding data were more likely to be younger with lower socio-economic status, factors which have repeatedly been shown to be associated with lower rates of breast-feeding initiation and continuation^(34)^. Because global breast-feeding rates vary substantially^(58)^, the analysis of all non-Canadian-born participants as a single group may have affected the findings. Furthermore, variations in participant recruitment practices at different sites many limit the conclusions drawn from the regional differences in breast-feeding practices. In Vancouver, participants were recruited from a single site and all resided in the Lower Mainland of British Columbia, while in Winnipeg, participants were recruited either in Winnipeg or in a rural location in the province of Manitoba. Lastly, in an observational study, we may not be able to fully account for unmeasured confounders.

## Conclusion

In the CHILD birth cohort, Canadian-born and non-Canadian-born women had similar breast-feeding initiation and continuation rates. Our findings suggest that rates of breast-feeding initiation and continuation in Canadian-born women are more strongly associated with socio-demographic characteristics than in non-Canadian-born women. Place of residence continues to be highly predictive of breast-feeding continuation in both groups of women, and further research is required to identify the causes of these disparities and to develop strategies to improve breast-feeding rates nationally.

## Supplementary material

For supplementary material accompanying this paper visit https://doi.org/10.1017/S1368980021004699. click here to view supplementary material

## References

[1] Chowdhury R , Sinha B , Sankar MJ et al. (2015) Breastfeeding and maternal health outcomes: a systematic review and meta-analysis. Acta Paediatr 104, 96–113. 26172878 10.1111/apa.13102 PMC4670483

[2] Victora CG , Bahl R , Barros AJD et al. (2016) Breastfeeding in the 21st century: epidemiology, mechanisms, and lifelong effect. Lancet 387, 475–490. 26869575 10.1016/S0140-6736(15)01024-7

[3] Lodge CJ , Tan DJ , Lau MX et al. (2015) Breastfeeding and asthma and allergies: a systematic review and meta-analysis. Acta Paediatr 104, 38–53. 10.1111/apa.13132 26192405

[4] Azad MB , Vehling L , Lu Z et al. (2017) Breastfeeding, maternal asthma and wheezing in the first year of life: a longitudinal birth cohort study. Eur Respir J 49, 1602019. 28461293 10.1183/13993003.02019-2016

[5] Gibbs BG , Forste R & Lybbert E (2018) Breastfeeding, parenting, and infant attachment behaviors. Matern Child Health J 22, 579–588. 29388115 10.1007/s10995-018-2427-z

[6] Gionet L (2013) Breastfeeding Trends in Canada. https://www150.statcan.gc.ca/n1/pub/82-624-x/2013001/article/11879-eng.htm (accessed February 2020).

[7] Lok KYW , Wang MP , Chan VHS et al. (2018) Effect of secondary cigarette smoke from household members on breastfeeding duration: a prospective cohort study. Breastfeed Med 13, 412–417. 29902073 10.1089/bfm.2018.0024

[8] Bai DL , Fong DY & Tarrant M (2015) Factors associated with breastfeeding duration and exclusivity in mothers returning to paid employment postpartum. Matern Child Health J 19, 990–999. 25095769 10.1007/s10995-014-1596-7

[9] Tavoulari EF , Benetou V , Vlastarakos PV et al. (2015) Immigrant status as important determinant of breastfeeding practice in southern Europe. Cent Eur J Public Health 23, 39–44. 26036097 10.21101/cejph.a4092

[10] Bai DL , Wu KM & Tarrant M (2013) Association between intrapartum interventions and breastfeeding duration. J Midwifery Wom Heal 58, 25–32. 10.1111/j.1542-2011.2012.00254.x 23317341

[11] Chiang KV , Sharma AJ , Nelson JM et al. (2019) Receipt of breast milk by gestational age – United States, 2017. MMWR Morb Mortal Wkly Rep 68, 489–493. 31170123 10.15585/mmwr.mm6822a1 PMC6553805

[12] Vehling L , Chan D , McGavock J et al. (2018) Exclusive breastfeeding in hospital predicts longer breastfeeding duration in Canada: implications for health equity. Birth 45, 440–449. 29498088 10.1111/birt.12345

[13] Hobbs AJ , Mannion CA , McDonald SW et al. (2016) The impact of caesarean section on breastfeeding initiation, duration and difficulties in the first four months postpartum. BMC Pregnancy Childbirth 16, 90. 27118118 10.1186/s12884-016-0876-1 PMC4847344

[14] DiGirolamo AM , Grummer-Strawn LM & Fein SB (2008) Effect of maternity-care practices on breastfeeding. Pediatrics 122, S43–49. 18829830 10.1542/peds.2008-1315e

[15] Ladewig EL , Hayes C , Browne J et al. (2014) The influence of ethnicity on breastfeeding rates in Ireland: a cross-sectional study. J Epidemiol Community Health 68, 356–362. 24336238 10.1136/jech-2013-202735

[16] Anstey EH , Chen J , Elam-Evans LD et al. (2017) Racial and geographic differences in breastfeeding – United States, 2011–2015. MMWR Morb Mortal Wkly Rep 66, 723–727. 28704352 10.15585/mmwr.mm6627a3 PMC5687589

[17] Dennis CL , Shiri R , Brown HK et al. (2019) Breastfeeding rates in immigrant and non-immigrant women: a systematic review and meta-analysis. Matern Child Nutr 15, e12809. 30884175 10.1111/mcn.12809 PMC7199026

[18] Lok KYW , Bai DL , Chan NPT et al. (2018) The impact of immigration on the breastfeeding practices of Mainland Chinese immigrants in Hong Kong. Birth 45, 94–102. 28960460 10.1111/birt.12314

[19] Aguilar-Ortega JM , Gonzalez-Pascual JL , Cardenete-Reyes C et al. (2019) Adherence to initial exclusive breastfeeding among Chinese born and native Spanish mothers. BMC Pregnancy Childbirth 19, 44. 30691401 10.1186/s12884-018-2161-y PMC6348660

[20] Schmied V , Olley H , Burns E et al. (2012) Contradictions and conflict: a meta-ethnographic study of migrant women’s experiences of breastfeeding in a new country. BMC Pregnancy Childbirth 12, 163. 23270315 10.1186/1471-2393-12-163 PMC3546887

[21] Hendrick CE & Potter JE (2017) Nativity, country of education, and Mexican-Origin women’s breastfeeding behaviors in the first 10 months postpartum. Birth 44, 68–77. 27779318 10.1111/birt.12261 PMC5654533

[22] Statistics Canada (2017) Study: Women in Canada: Women and Paid Work. Women in Canada: A Gender-Based Statistical Report, 7th ed. Ottawa, ON: Statistics Canada.

[23] Hudon T (2015) Women in Canada: A Gender-Based Statistical Report. Immigrant Women. Ottawa: Statistics Canada.

[24] Hawkins SS , Gillman MW , Shafer EF et al. (2014) Acculturation and maternal health behaviors: findings from the Massachusetts birth certificate. Am J Prev Med 47, 150–159. 24951043 10.1016/j.amepre.2014.02.015 PMC4106991

[25] Subbarao P , Anand SS , Becker AB et al. (2015) The Canadian Healthy Infant Longitudinal Development (CHILD) Study: examining developmental origins of allergy and asthma. Thorax 70, 998–1000. 26069286 10.1136/thoraxjnl-2015-207246

[26] World Health Organization & UNICEF (2009) Baby-Friendly Hospital Initiative: Revised, Updated and Expanded for Integrated Care. Geneva: World Health Organization. 23926623

[27] World Health Organization (2008) Indicators for Assessing Infant and Young Child Feeding Practices. Geneva: World Health Organization.

[28] The World Bank (2019) World Bank Country and Lending Groups. https://datahelpdesk.worldbank.org/knowledgebase/articles/906519-world-bank-country-and-lending-groups (accessed February 2020).

[29] United Nations Development Programme (2019) Human Development Indices and Indicators: 2018 Statistical Update. Geneva: United Nations Development Programme.

[30] Williams TC , Bach CC , Matthiesen NB et al. (2018) Directed acyclic graphs: a tool for causal studies in paediatrics. Pediatr Res 84, 487–493. 29967527 10.1038/s41390-018-0071-3 PMC6215481

[31] StataCorp (2017) Stata Statistical Software: Release 15.1. College Station, TX: StataCorp LP.

[32] Tavoulari EF , Benetou V , Vlastarakos PV et al. (2015) Factors affecting breast-feeding initiation in Greece: what is important? Midwifery 31, 323–331. 25467601 10.1016/j.midw.2014.10.006

[33] Thulier D & Mercer J (2009) Variables associated with breastfeeding duration. J Obstet Gynecol Neonatal Nurs 38, 259–268. 10.1111/j.1552-6909.2009.01021.x 19538614

[34] Cohen SS , Alexander DD , Krebs NF et al. (2018) Factors associated with breastfeeding initiation and continuation: a meta-analysis. J Pediatr 203, 190–196.e121. 30293638 10.1016/j.jpeds.2018.08.008

[35] Tarrant M , Fong DY , Wu KM et al. (2010) Breastfeeding and weaning practices among Hong Kong mothers: a prospective study. BMC Pregnancy Childbirth 10, 27. 20509959 10.1186/1471-2393-10-27 PMC2887376

[36] Zhao J , Zhao Y , Du M et al. (2017) Maternal education and breastfeeding practices in China: a systematic review and meta-analysis Midwifery 50, 62–71. 28390256 10.1016/j.midw.2017.03.011

[37] Bulk-Bunschoten AM , Pasker-de Jong PC , van Wouwe JP et al. (2008) Ethnic variation in infant-feeding practices in the Netherlands and weight gain at 4 months. J Hum Lact 24, 42–49. 18281355 10.1177/0890334407311338

[38] Green M , Chalmers B , Hanvey L et al. (2019) Breastfeeding. In Family-Centred Maternity and Newborn Care: National Guidelines, pp. 6.1–6.54 [Public Health Agency of Canada, editor]. Ottawa, ON: Public Health Agency of Canada.

[39] Tarrant M , Lok KY , Fong DY et al. (2016) Effect on baby-friendly hospital steps when hospitals implement a policy to pay for infant formula. J Hum Lact 32, 238–249. 26286469 10.1177/0890334415599399

[40] Brodribb W , Kruske S & Miller YD (2013) Baby-friendly hospital accreditation, in-hospital care practices, and breastfeeding. Pediatrics 131, 685–692. 23478863 10.1542/peds.2012-2556

[41] Horta BL , Kramer MS & Platt RW (2001) Maternal smoking and the risk of early weaning: a meta-analysis. Am J Public Health 91, 304–307. 11211645 10.2105/ajph.91.2.304 PMC1446540

[42] Napierala M , Mazela J , Merritt TA et al. (2016) Tobacco smoking and breastfeeding: effect on the lactation process, breast milk composition and infant development. A critical review. Environ Res 151, 321–338. 27522570 10.1016/j.envres.2016.08.002

[43] Baron JA , Bulbrook RD , Wang DY et al. (1986) Cigarette smoking and prolactin in women. Br Med J 293, 482–483. 3790209 10.1136/bmj.293.6545.482 PMC1341114

[44] Lok KY , Bai DL & Tarrant M (2015) Predictors of breastfeeding initiation in Hong Kong and Mainland China born mothers. BMC Pregnancy Childbirth 15, 286. 26531299 10.1186/s12884-015-0719-5 PMC4632339

[45] Donath SM , Amir LH & Team AS (2004) The relationship between maternal smoking and breastfeeding duration after adjustment for maternal infant feeding intention. Acta Paediatr 93, 1514–1518. 15513582 10.1080/08035250410022125

[46] Bai DL , Fong D , Lok KYW et al. (2016) Relationship between the Infant Feeding preferences of Chinese mother’s immediate social network and early breastfeeding cessation. J Hum Lact 32, 301–308. 26887843 10.1177/0890334416630537

[47] Lok KYW , Bai DL & Tarrant M (2017) Family members’ infant feeding preferences, maternal breastfeeding exposures and exclusive breastfeeding intentions. Midwifery 53, 49–54. 28755584 10.1016/j.midw.2017.07.003

[48] Prior E , Santhakumaran S , Gale C et al. (2012) Breastfeeding after cesarean delivery: a systematic review and meta-analysis of world literature. Am J Clin Nutr 95, 1113–1135. 22456657 10.3945/ajcn.111.030254

[49] Fan HSL , Wong JYH , Fong DYT et al. (2019) Association between early-term birth and breastfeeding initiation, duration, and exclusivity: a systematic review. Birth 46, 24–34. 30051544 10.1111/birt.12380

[50] Fan HSL , Wong JYH , Fong DYT et al. (2019) Breastfeeding outcomes among early-term and full-term infants. Midwifery 71, 71–76. 30690202 10.1016/j.midw.2019.01.005

[51] Sengupta S , Carrion V , Shelton J et al. (2013) Adverse neonatal outcomes associated with early-term birth. JAMA Pediatr 167, 1053–1059. 24080985 10.1001/jamapediatrics.2013.2581

[52] Tita AT , Landon MB , Spong CY et al. (2009) Timing of elective repeat cesarean delivery at term and neonatal outcomes. NEJM 360, 111–120. 19129525 10.1056/NEJMoa0803267 PMC2811696

[53] Rio I , Castello-Pastor A , Del Val Sandin-Vazquez M et al. (2011) Breastfeeding initiation in immigrant and non-immigrant women in Spain. Eur J Clin Nutr 65, 1345–1347. 21712837 10.1038/ejcn.2011.121

[54] Barcelona de Mendoza V , Harville E , Theall K et al. (2016) Acculturation and intention to breastfeed among a population of predominantly Puerto Rican Women. Birth 43, 78–85. 26554873 10.1111/birt.12199 PMC4755899

[55] Harley K , Stamm NL & Eskenazi B (2007) The effect of time in the U.S. on the duration of breastfeeding in women of Mexican descent. Matern Child Health J 11, 119–125. 17279324 10.1007/s10995-006-0152-5 PMC3957412

[56] Dennis CL , Gagnon A , Van Hulst A et al. (2014) Predictors of breastfeeding exclusivity among migrant and Canadian-born women: results from a multi-centre study. Matern Child Nutr 10, 527–544. 22974539 10.1111/j.1740-8709.2012.00442.x PMC6860320

[57] Statistics Canada (2017) Education in Canada: Key results from the 2016 Census. https://www150.statcan.gc.ca/n1/en/daily-quotidien/171129/dq171129a-eng.pdf?st=YiBkCOOc (accessed September 2020).

[58] United Nations Children’s Fund (UNICEF) (2018) Breastfeeding: A Mother’s Gift, for Every Child. New York: UNICEF.

