# Supplementary material for: Factors associated with breast-feeding initiation and continuation in Canadian-born and non-Canadian-born women: a multi-centre study
Source: Public Health Nutr. 2021 Dec 3;25(10):2822–33. doi: 10.1017/S1368980021004699 (PMC9991853; doi:10.1017/S1368980021004699)
Supplement: Supplementary file 1 [file S1368980021004699sup001.docx]

| **Table S1.** A comparison of the characteristics of study participants by follow-up status (n=3,264) | | | | |
| --- | --- | --- | --- | --- |
| Characteristic | No relevant missing data  N=2,906  n (%) | Missing data*  N=254  n (%) | Lost to follow-up before 6 months  N=104  n (%) | *p*-value |
| Maternal age (years) |  |  |  | <.001 |
| 18 – 24 | 195 (6.7) | 38 (15.0) | 4 (3.9) |  |
| 25 – 29 | 649 (22.3) | 69 (27.3) | 22 (21.2) |  |
| 30 – 34 | 1,243 (42.8) | 85 (33.6) | 44 (42.3) |  |
| ≥ 35 | 819 (28.2) | 61 (24.1) | 34 (32.7) |  |
| Maternal education |  |  |  | .001 |
| High-school or below | 247 (8.5) | 23 (18.4) | 7 (6.7) |  |
| Some postsecondary | 833 (28.7) | 43 (34.4) | 30 (28.9) |  |
| University degree or above | 1,826 (62.8) | 59 (47.2) | 67 (64.4) |  |
| Annual household income (CAD) |  |  |  | <.001 |
| < $50,000 | 354 (12.2) | 26 (10.2) | 20 (19.2) |  |
| $50,000 – $99,999 | 889 (30.6) | 33 (13.0) | 21 (20.2) |  |
| $100,000 – $149,999 | 745 (25.6) | 26 (10.2) | 24 (23.1) |  |
| ≥ $150,000 | 644 (22.2) | 27 (10.6) | 29 (27.9) |  |
| Not specified | 274 (9.4) | 142 (55.9) | 10 (9.6) |  |
| Home ownership |  |  |  | <.001 |
| No | 747 (25.7) | 174 (68.5) | 36 (34.6) |  |
| Yes | 2,159 (74.3) | 80 (31.5) | 68 (65.4) |  |
| Married or living with partner |  |  |  | <.001 |
| No | 175 (6.0) | 116 (45.7) | 8 (7.7) |  |
| Yes | 2,731 (94.0) | 138 (54.3) | 96 (92.3) |  |
| Study recruitment site |  |  |  | <.001 |
| Edmonton | 666 (22.9) | 82 (32.3) | 17 (16.4) |  |
| Toronto | 673 (23.2) | 42 (16.5) | 50 (48.1) |  |
| Vancouver | 662 (22.8) | 47 (18.5) | 21 (20.2) |  |
| Winnipeg | 905 (31.1) | 83 (32.7) | 16 (15.4) |  |
| *Numbers do not always add up to N because of missing values | | | | |

| **Table S2.** Unadjusted odds ratios (OR) for breastfeeding initiation and any breastfeeding at 6 months postpartum by migration factors in non-Canadian Born participants | | | | |
| --- | --- | --- | --- | --- |
|  | Breastfeeding Initiation (n=739) | | Any Breastfeeding at 6-months (n=692) | |
| Characteristic | OR | (95% CI) | OR | (95% CI) |
| Years lived in Canada |  |  |  |  |
| < 5 | 1 | -- | 1 | -- |
| 5 - <10 | 1.38 | 0.50 **–** 3.81 | 0.99 | 0.57 **–** 1.71 |
| 10 - <20 | 2.04 | 0.64 **–** 6.54 | 1.07 | 0.61 **–** 1.88 |
| ≥ 20 | 0.92 | 0.40 **–** 2.13 | 0.81 | 0.50 **–** 1.33 |
| Age at migration |  |  |  |  |
| <18 years | 1 | -- | 1 | -- |
| => 18 years | 1.45 | 0.72 – 2.92 | 1.14 | 0.78 **–** 1.69 |
| Gender Development Index |  |  |  |  |
| High | 1 | -- | 1 | -- |
| Moderate | 1.33 | 0.64 – 2.77 | 1.22 | 0.81 **–** 1.83 |
| Low | 6.29 | 0.83 – 47.4 | 1.44 | 0.76 – 2.69 |
| Geographic region |  |  |  |  |
| North America | 1 | -- | 1 | -- |
| Latin America & Caribbean | 1.19 | 0.31 – 4.61 | 0.57 | 0.24 **–** 1.32 |
| Europe & Central Asia | 1.37 | 0.40 – 4.68 | 0.76 | 0.34 **–** 1.70 |
| Middle East & Africa | 2.15 | 0.38 – 12.1 | 0.56 | 0.22 **–** 1.41 |
| Asia & Pacific | 1.21 | 0.39 – 3.80 | 0.63 | 0.29 **–** 1.35 |
| Country income level |  |  |  |  |
| High | 1 | -- | 1 | -- |
| Upper middle | 1.23 | 0.52 – 2.93 | 1.01 | 0.63 **–** 1.63 |
| Lower middle - low | 1.19 | 0.52 – 2.75 | 0.75 | 0.48 **–** 1.17 |

| **Table S3.** Unadjusted and adjusted odds ratios for any breastfeeding at six months postpartum by birthplace among only participants who initiated breastfeeding (n=2,707) | | | | | | | | |
| --- | --- | --- | --- | --- | --- | --- | --- | --- |
|  | Canadian Born Participants (n=2,048) | | | | Non-Canadian Born Participants (n=659) | | | |
| Characteristic | OR | (95% CI) | ^a^OR | (95% CI) | OR | (95% CI) | ^a^OR | (95% CI) |
| ***Sociodemographic characteristics*** | | | | | | | | |
| Maternal age (years) |  |  |  |  |  |  |  |  |
| 18 – 24 | 1 | -- | -- | -- | 1 | -- | -- | -- |
| 25 – 29 | 3.78 | 2.50 – 5.71 | -- | -- | 4.25 | 1.67 – 10.8 | -- | -- |
| 30 – 34 | 4.50 | 3.06 – 6.62 | -- | -- | 2.93 | 1.28 – 6.78 | -- | -- |
| ≥ 35 | 6.70 | 4.37 – 10.3 | -- | -- | 3.05 | 1.30 – 7.14 | -- | -- |
| Maternal education |  |  |  |  |  |  |  |  |
| High-school or below | 1 | -- | 1 | -- | 1 | -- | 1 | -- |
| Some post-secondary | 1.82 | 1.26 – 2.62 | 1.47 | 1.00 – 2.16 | 0.93 | 0.39 – 1.21 | 0.67 | 0.25 – 1.78 |
| University degree or above | 4.28 | 2.99 – 6.11 | 2.92 | 1.97 – 4.34 | 1.94 | 0.85 – 4.45 | 1.39 | 0.53 – 3.65 |
| Annual household income (CAD) |  |  |  |  |  |  |  |  |
| < $50,000 | 1 | -- | 1 | -- | 1 | -- | 1 | -- |
| $50,000 ‒ $99,999 | 2.28 | 1.58 – 3.30 | 1.22 | 0.81 – 1.85 | 1.16 | 0.62 – 2.15 | 1.04 | 0.53 – 2.03 |
| $100,000 ‒ $149,999 | 2.24 | 1.55 – 3.24 | 0.91 | 0.58 – 1.42 | 0.93 | 0.48 – 1.83 | 0.63 | 0.30 – 1.36 |
| ≥ $150,000 | 2.56 | 1.73 – 3.78 | 0.89 | 0.54 – 1.45 | 1.11 | 0.56 – 2.22 | 0.77 | 0.34 – 1.74 |
| Not specified | 1.84 | 1.16 – 2.95 | 1.26 | 0.76 – 2.09 | 1.55 | 0.61 – 3.92 | 2.03 | 0.74 – 5.54 |
| Home ownership |  |  |  |  |  |  |  |  |
| No | 1 | -- | 1 | -- | 1 | -- | 1 | -- |
| Yes | 1.35 | 1.04 – 1.75 | 1.01 | 0.74 – 1.39 | 1.40 | 0.91 – 2.17 | 1.58 | 0.96 – 2.60 |
| Married or living with partner |  |  |  |  |  |  |  |  |
| No | 1 | -- | 1 | -- | 1 | -- | 1 | -- |
| Yes | 3.21 | 2.17 – 4.75 | 1.79 | 1.16 – 2.77 | 2.37 | 1.02 – 5.55 | 1.76 | 0.72 – 4.31 |
| Smoked during pregnancy |  |  |  |  |  |  |  |  |
| No | 1 | -- | 1 | -- | 1 | -- | 1 | -- |
| Yes | 0.21 | 0.13 – 0.33 | 0.42 | 0.25 – 0.72 | 0.07 | 0.12 – 0.35 | 0.08 | 0.01 – 0.46 |
| Living with current smoker |  |  |  |  |  |  |  |  |
| No | 1 | -- | 1 | -- | 1 | -- | 1 | -- |
| Yes | 0.39 | 0.29 – 0.52 | 0.56 | 0.41 – 0.77 | 0.60 | 0.32 – 1.12 | 0.71 | 0.36 – 1.37 |
|  |  |  |  |  |  |  |  |  |
|  |  |  |  |  |  |  |  |  |
|  |  |  |  |  |  |  |  |  |
| Study recruitment site |  |  |  |  |  |  |  |  |
| Edmonton | 1 | -- | -- | -- | 1 | -- | -- | -- |
| Toronto | 1.23 | 0.90 – 1.70 | -- | -- | 1.71 | 0.94 – 3.12 | -- | -- |
| Vancouver | 2.61 | 1.78 – 3.84 | -- | -- | 2.38 | 1.31 – 4.33 | -- | -- |
| Winnipeg | 1.03 | 0.78 – 1.36 | -- | -- | 1.66 | 0.87 – 3.17 | -- | -- |
| *Maternal and Birth Characteristics* | | | | | | | | |
| Parity |  |  |  |  |  |  |  |  |
| Primiparous | 1 | -- | 1 | -- | 1 | -- | 1 | -- |
| Multiparous | 1.21 | 0.97 – 1.52 | 1.14 | 0.90 – 1.46 | 0.85 | 0.55 – 1.30 | 0.91 | 0.57 – 1.45 |
| Mode of birth |  |  |  |  |  |  |  |  |
| Vaginal | 1 | -- | 1 | -- | 1 | -- | 1 | -- |
| Cesarean | 0.68 | 0.53 – 0.87 | 0.62 | 0.48 – 0.82 | 0.84 | 0.53 – 1.34 | 0.79 | 0.48 – 1.31 |
| Gestational age |  |  |  |  |  |  |  |  |
| Full-term (≥ 39 weeks) | 1 | -- | 1 | -- | 1 | -- | 1 | -- |
| Early-term (37 – <39 weeks) | 0.71 | 0.54 – 0.91 | 0.70 | 0.53 – 0.91 | 0.81 | 0.50 – 1.31 | 0.89 | 0.54 – 1.47 |
| Preterm (35 – <37 weeks) | 0.86 | 0.51 – 1.47 | 0.89 | 0.51 – 1.56 | 1.12 | 0.32 – 3.87 | 1.54 | 0.39 – 6.14 |
| Birth weight (grams) |  |  |  |  |  |  |  |  |
| < 2,500 | 1 | -- | 1 | -- | 1 | -- | 1 | -- |
| 2,500 – < 3,250 | 1.28 | 0.61 – 2.67 | 1.26 | 0.57 – 2.82 | 0.32 | 0.04 – 2.48 | 0.36 | 0.04 – 3.02 |
| 3,250 – < 4,000 | 1.32 | 0.64 – 2.72 | 1.18 | 0.52 – 2.66 | 0.22 | 0.03 – 1.65 | 0.24 | 0.03 – 1.99 |
| ≥ 4,000 | 1.18 | 0.55 – 2.53 | 0.98 | 0.41 – 2.31 | 0.35 | 0.04 – 2.98 | 0.33 | 0.04 – 3.13 |
| Baby rooming in with mother |  |  |  |  |  |  |  |  |
| No | 1 | -- | 1 | -- | 1 | -- | 1 | -- |
| Yes | 0.94 | 0.69 – 1.28 | 0.94 | 0.69 – 1.28 | 0.97 | 0.53 – 1.80 | 1.01 | 0.54 – 1.88 |

^a^ covariates for each model are as follows:

Maternal Age: no adjustment needed

Maternal Education: adjusted for maternal age

Household Income: adjusted for maternal age, education, marital status and study site

Home Ownership: adjusted for maternal age, education, marital status, household income, study site

Marital Status: adjusted for maternal age and education

Smoked During Pregnancy: adjusted for maternal age, education, marital status, household income, live with current smoker

Live with Current Smoker: adjusted for household income and maternal education

Study Recruitment Site: no adjustment needed

Parity: adjusted for maternal age, education, marital status, household income

Mode of Birth: adjusted for maternal age, education, household income, smoked during pregnancy, live with a current smoker, parity, gestational age, and birth weight

Gestational age: adjusted for maternal age, education, household income, smoked during pregnancy, live with current smoker, and parity

Birth weight: adjusted for maternal age, education, household income, smoked during pregnancy, live with current smoker, parity and gestational age

Baby Rooming in with Mother: adjusted for mode of birth, gestational age and birth weight

| **Table S4.** Basic demographic characteristics of study participants by place of birth and study site | | | | | | | | |
| --- | --- | --- | --- | --- | --- | --- | --- | --- |
|  | Non-Canadian Born Participants (n=739) | | | | Canadian Born Participants (n=2271) | | | |
|  | Edmonton | Toronto | Vancouver | Winnipeg | Edmonton | Toronto | Vancouver | Winnipeg |
| Characteristic | N=124  N (%) | N=215  N (%) | N=253  N (%) | N=147  N (%) | N=559  N (%) | N=508  N (%) | N=430  N (%) | N=774  N (%) |
| ***Sociodemographic characteristics*** | | | | | | | | |
| Maternal age (years) | | | | | | | | |
| 18 – 24 | 7 (5.7) | 7 (3.3) | 2 (0.8) | 17 (11.6) | 40 (7.2) | 7 (1.4) | 4 (0.9) | 115 (14.9) |
| 25 – 29 | 37 (29.8) | 30 (14.0) | 39 (15.4) | 52 (35.4) | 169 (30.2) | 67 (13.2) | 52 (12.1) | 225 (29.1) |
| 30 – 34 | 53 (42.7) | 96 (44.7) | 98 (38.7) | 59 (40.1) | 238 (42.6) | 242 (47.6) | 183 (42.6) | 318 (41.1) |
| ≥ 35 | 27 (21.8) | 82 (38.1) | 114 (45.1) | 19 (12.9) | 112 (20.0) | 192 (37.8) | 191 (44.4) | 116 (15.0) |
| Maternal education |  |  |  |  |  |  |  |  |
| High-school or below | 7 (5.7) | 9 (4.2) | 4 (1.6) | 28 (19.1) | 46 (8.2) | 13 (2.6) | 13 (3.0) | 134 (17.3) |
| Some post-secondary | 42 (33.9) | 39 (18.1) | 66 (26.1) | 33 (22.5) | 225 (40.3) | 92(18.1) | 87 (20.2) | 279 (36.1) |
| University degree or above | 75 (60.5) | 167 (77.7) | 183 (72.3) | 86 (58.5) | 288 (51.5) | 403 (79.3) | 330 (76.7) | 361 (46.6) |
| Annual household income (CAD) |  |  |  |  |  |  |  |  |
| < $50,000 | 22 (17.7) | 20 (9.3) | 43 (17.0) | 55 (37.4) | 51 (9.1) | 12 (2.4) | 22 (5.1) | 149 (19.3) |
| $50,000 ‒ $99,999 | 50 (40.3) | 50 (23.3) | 83 (32.8) | 56 (38.1) | 174 (31.1) | 74 (14.6) | 126 (29.3) | 297 (38.4) |
| $100,000 ‒ $149,999 | 17 (13.7) | 45 (20.9) | 62 (24.5) | 17 (11.6) | 191 (34.2) | 130 (25.6) | 137 (31.9) | 170 (22.0) |
| ≥ $150,000 | 18 (14.5) | 75 (34.9) | 44 (17.4) | 4 (2.7) | 114 (20.4) | 231 (45.5) | 125 (29.1) | 62 (8.0) |
| Not specified | 17 (13.7) | 25 (11.6) | 21 (8.3) | 15 (10.2) | 29 (5.2) | 61 (12.0) | 20 (4.7) | 96 (12.4) |
| Home ownership |  |  |  |  |  |  |  |  |
| No | 45 (36.3) | 67 (32.2) | 117 (46.3) | 51 (34.7) | 108 (19.3) | 94 (18.5) | 153 (35.6) | 148 (19.1) |
| Yes | 79 (63.7) | 148 (68.8) | 136 (53.8) | 96 (65.3) | 451 (80.7) | 414 (81.5) | 277 (64.4) | 626 (80.9) |
| Married or living with partner |  |  |  |  |  |  |  |  |
| No | 8 (6.5) | 13 (6.1) | 10 (4.0) | 6 (4.1) | 31 (5.6) | 24 (4.7) | 15 (3.5) | 76 (9.8) |
| Yes | 116 (93.6) | 202 (94.0) | 243 (96.1) | 141 (95.9) | 528 (94.5) | 484 (95.3) | 415 (96.5) | 698 (90.2) |

| **Table S5.** Migration characteristics of non-Canadian born study participants by study site | | | | |
| --- | --- | --- | --- | --- |
|  | Edmonton | Toronto | Vancouver | Winnipeg |
| Characteristic | N=124  N (%) | N=215  N (%) | N=253  N (%) | N=147  N (%) |
| Years lived in Canada |  |  |  |  |
| < 5 | 46 (37.1) | 39 (18.1) | 76 (30.0) | 52 (35.4) |
| 5 - <10 | 19 (15.3) | 46 (21.4) | 59 (23.3) | 34 (23.1) |
| 10 - <20 | 26 (21.0) | 43 (20.0) | 59 (23.3) | 26 (17.7) |
| ≥ 20 | 33 (26.6) | 87 (40.5) | 59 (23.3) | 35 (23.8) |
| Age at migration |  |  |  |  |
| <18 years | 49 (39.5) | 104 (48.4) | 69 (27.3) | 51 (34.7) |
| => 18 years | 75 (60.5) | 111 (51.6) | 184 (72.7) | 96 (65.3) |
| GDI |  |  |  |  |
| High | 54 (43.6) | 117 (54.4) | 126 (49.8) | 57 (38.8) |
| Moderate | 41 (33.1) | 68 (31.6) | 106 (41.9) | 64 (43.5) |
| Low | 29 (23.4) | 30 (14.0) | 21 (8.3) | 26 (17.7) |
| Geographic region |  |  |  |  |
| North America | 8 (6.5) | 15 (7.0) | 31 (12.3) | 17 (11.6) |
| Latin America & Caribbean | 15 (12.1) | 28 (13.0) | 27 (10.7) | 35 (23.8) |
| Europe & Central Asia | 29 (23.4) | 71 (33.0) | 69 (27.3) | 22 (15.0) |
| Middle East & Africa | 14 (11.3) | 34 (15.8) | 15 (5.9) | 11 (7.5) |
| Asia & Pacific | 58 (46.8) | 67 (31.2) | 111 (43.9) | 62 (42.2) |
| Country income level |  |  |  |  |
| High | 49 (39.5) | 94 (43.7) | 142 (56.1) | 40 (27.2) |
| Upper middle | 24 (19.4) | 61 (28.4) | 56 (22.1) | 57 (38.8) |
| Lower middle - low | 51 (41.1) | 60 (27.9) | 55 (21.7) | 50 (34.0) |
